# Rethinking the assessment of risk of bias due to selective reporting: a cross-sectional study

**DOI:** 10.1186/s13643-016-0289-2

**Published:** 2016-07-08

**Authors:** Matthew J. Page, Julian P. T. Higgins

**Affiliations:** School of Social and Community Medicine, University of Bristol, Canynge Hall, 39 Whatley Road, Bristol, BS8 2PS UK; School of Public Health and Preventive Medicine, Monash University, Level 6, The Alfred Centre, 99 Commercial Road, Melbourne, VIC 3004 Australia

**Keywords:** Bias, Quality, Methodology, Randomised trials, Systematic reviews

## Abstract

**Background:**

Selective reporting is included as a core domain of Cochrane’s tool for assessing risk of bias in randomised trials. There has been no evaluation of review authors’ use of this domain. We aimed to evaluate assessments of selective reporting in a cross-section of Cochrane reviews and to outline areas for improvement.

**Methods:**

We obtained data on selective reporting judgements for 8434 studies included in 586 Cochrane reviews published from issue 1–8, 2015. One author classified the reasons for judgements of high risk of selective reporting bias. We randomly selected 100 reviews with at least one trial rated at high risk of outcome non-reporting bias (non-/partial reporting of an outcome on the basis of its results). One author recorded whether the authors of these reviews incorporated the selective reporting assessment when interpreting results.

**Results:**

Of the 8434 studies, 1055 (13 %) were rated at high risk of bias on the selective reporting domain. The most common reason was concern about outcome non-reporting bias. Few studies were rated at high risk because of concerns about bias in selection of the reported result (e.g. reporting of only a subset of measurements, analysis methods or subsets of the data that were pre-specified). Review authors often specified in the risk of bias tables the study outcomes that were not reported (84 % of studies) but less frequently specified the outcomes that were partially reported (61 % of studies). At least one study was rated at high risk of outcome non-reporting bias in 31 % of reviews. In the random sample of these reviews, only 30 % incorporated this information when interpreting results, by acknowledging that the synthesis of an outcome was missing data that were not/partially reported.

**Conclusions:**

Our audit of user practice in Cochrane reviews suggests that the assessment of selective reporting in the current risk of bias tool does not work well. It is not always clear which outcomes were selectively reported or what the corresponding risk of bias is in the synthesis with missing outcome data. New tools that will make it easier for reviewers to convey this information are being developed.

**Electronic supplementary material:**

The online version of this article (doi:10.1186/s13643-016-0289-2) contains supplementary material, which is available to authorized users.

## Background

Reports of randomised trials should provide a complete and balanced account of the findings. However, many reports are plagued by selective reporting [1]. One type of selective reporting, which we call “outcome non-reporting bias”, occurs when some outcomes that were measured and analysed are not reported or are partially reported based on the nature of the results (e.g. statistical significance or magnitude of effect) [2]. For example, participant deaths may be counted and compared between intervention groups but trialists present no data because the effect favoured the comparator or only state that the between-group difference was not statistically significant; in this case, the summary statistics needed to include the trial in a meta-analysis are unavailable [3, 4]. Another type of selective reporting, which we call “bias in selection of the reported result”, occurs when the effect estimate that is fully reported in a publication has been selected from among multiple measurements or analyses (e.g. trialists perform multiple adjusted analyses yet only report that which yielded the most favourable effect estimate) [5]. Given the frequency with which both types of selective reporting occur [4, 6, 7], authors of systematic reviews are encouraged to assess these sources of bias in the included studies.

Selective reporting is included as one of the core domains of the Cochrane tool for assessing the risk of bias in randomised trials (RoB tool) [8]. The domain was included in the tool when it was created in 2006, in response to emerging evidence of worrying degrees of selective reporting [3, 9]; at that time, no existing risk of bias tool addressed the issue. Review authors were asked to judge the risk of selective reporting bias as either low risk, high risk or unclear risk and to provide reasons for their judgements. Guidance for a judgement of high risk of bias, as specified in the Cochrane Handbook [10], is presented in Table 1. These criteria include examples of outcome non-reporting bias and bias in selection of the reported result.

**Table 1:** Criteria for a judgement of high risk of bias due to selective reporting in the Cochrane risk of bias tool for randomised trials (2011 version)

Any one of the following: (1) not all of the study’s pre-specified primary outcomes have been reported; (2) one or more outcomes of interest in the review are reported incompletely so that they cannot be entered in a meta-analysis; (3) the study report fails to include results for a key outcome that would be expected to have been reported for such a study; (4) one or more primary outcomes is reported using measurements, analysis methods or subsets of the data (e.g. subscales) that were not pre-specified, or; (5) one or more reported primary outcomes were not pre-specified (unless clear justification for their reporting is provided, such as an unexpected adverse event). We consider criteria 1–3 examples of outcome non-reporting bias and criteria 4–5 examples of bias in selection of the reported result.	

**Fig. 1:**
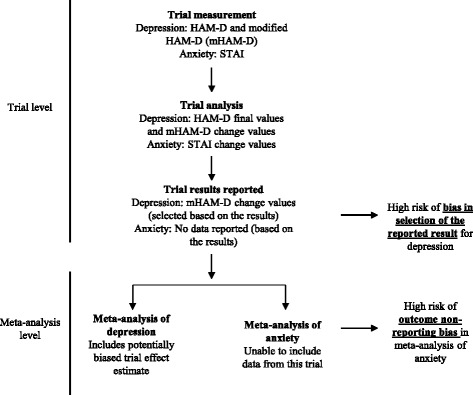
Distinction between outcome non-reporting bias and bias in selection of the reported result

Limitations of the assessment of selective reporting in the current RoB tool were recognised in an evaluation of the tool [11]. The problems arise mainly from the fact that assessments are conducted at the study-level, which has the following three implications:

1. All findings from a study are considered at high risk of bias on the basis that one or more outcomes are not/partially reported. However, it makes little sense to judge the fully reported outcomes in such trials at high risk of bias by default.
2. Review authors may judge a study at high risk of selective reporting bias but not declare in the RoB table the specific outcomes that were selectively reported (e.g. only state “Some outcomes were not reported”). This prevents readers from knowing which outcomes of the review should be interpreted with caution.
3. Outcome non-reporting bias and bias in selection of the reported result are considered simultaneously, which is not ideal because each has different consequences. Outcome non-reporting bias in one or more trials can put the treatment effect estimate of a *systematic review/meta-analysis* which cannot include the data at risk of bias [2, 12]. This is analogous to publication bias, whereby a whole study is inaccessible to review authors on the basis of the results. In contrast, bias in selection of the reported result puts effect estimates from *individual primary studies* at risk of bias in the same way as other domains in the RoB tool (e.g. attrition bias, detection bias), as well as putting the systematic review/meta-analytic effect estimate at risk of bias. An example of this distinction is presented in Fig. 1. In this example, there is a high risk of bias in selection of the reported result for depression because depression was measured and analysed in multiple ways, yet only the most favourable of all possible effect estimates was reported. Inclusion of this effect estimate can bias the corresponding meta-analysis of depression. In contrast, anxiety was measured and analysed but no data were reported because the results were unfavourable; this does not bias the trial, but it can lead to outcome non-reporting bias in the meta-analysis of anxiety which cannot include the unreported data from this trial. As another example, a trialist may measure blood glucose at 3 and 6 months, yet only report the 3-month data on the basis of its large, favourable result. In this instance, a meta-analysis of 3-month data includes trial data at high risk of bias in selection of the reported result, while a meta-analysis of 6-month data which cannot include the non-reported data from this trial is at high risk of outcome non-reporting bias.

Another limitation of the current RoB tool is that it lacks clear guidance on how to incorporate the assessment of outcome non-reporting bias into the interpretation a systematic review effect estimate.

To date, there has been no evaluation of Cochrane review authors’ use of the selective reporting domain in the current RoB tool. It is unclear how many studies are rated at low/unclear/high risk of selective reporting bias and what reasons are provided for the judgements; whether review authors specify the outcomes they suspect have been selectively reported, which readers need to determine which of the trial and review outcomes are problematic; and whether review authors acknowledge the risk of selective reporting bias in the synthesis when interpreting the results (e.g. state that a particular meta-analysis is missing studies with inaccessible outcome data).

The aim of this study is to evaluate the assessments of risk of bias due to selective reporting in a cross-section of Cochrane reviews, so as to inform the development of a revised tool.

## Methods

### Eligibility criteria

We included Cochrane reviews meeting the following criteria:

- review of a therapeutic or preventive intervention;
- published between issue 1 to 8, 2015, in the Cochrane Database of Systematic Reviews (CDSR) as a new, updated or amended review;
- included an assessment of selective reporting in the included studies using the RoB tool.

We excluded review protocols and reviews of methodology, diagnostic test accuracy and prognostic studies, because selective reporting is not a standard risk of bias domain in these reviews.

### Data source

All Cochrane reviews are prepared as RevMan files [13] which are stored in *Archie,* a database managed by the Cochrane Informatics and Knowledge Management Department (IKMD). In September 2015, the IKMD provided us with the selective reporting judgement (low risk, unclear risk or high risk) and supporting text for the judgement, extracted from *Archie*, for all studies in reviews meeting our eligibility criteria. The IKMD also provided the title, DOI, issue number, year of publication and Cochrane Review Group of each review. Data were supplied in a Microsoft Excel® file.

### Data extraction and classification

We categorised the supporting text of each trial rated at high risk of bias due to selective reporting. Text was initially classified under one of the five criteria specified in the Cochrane Handbook (outlined in the Table 1), or as “other” if it did not meet those criteria. New categories were subsequently generated for all “other” reasons using an iterative approach. That is, category labels were generated and sometimes amended when a new example was encountered, to ensure that all categories were mutually exclusive. Whenever a category label was amended, all previous classifications were reviewed and modified as appropriate.

In addition, we recorded the specific outcome(s) that were reported as having been selectively reported (e.g. “all-cause mortality”, “pain”, “adverse events”). Instances where review authors did not specify the outcome (e.g. only included a statement such as “Not all pre-specified outcomes were reported”) were recorded as “Not specified”.

We drew a random sample of 100 reviews with at least one trial rated at high risk of outcome non-reporting bias (i.e. non- or partial reporting of an outcome) using the random number generator in Microsoft Excel®. We extracted from each review the following: total number of included studies; number of studies at high risk of outcome non-reporting bias; specific outcomes rated at high risk of outcome non-reporting bias (as determined from the RoB tables); the reviewer-perceived importance of the outcome(s) at high risk of outcome non-reporting bias (i.e. “primary” or “secondary”); and whether a meta-analysis was performed on at least one of the outcome(s) at high risk of outcome non-reporting bias.

We then examined the main text (“Effect of interventions” section), abstract and Summary of Findings table of each review and recorded whether or not review authors acknowledged that a synthesis of an outcome was missing data that were not/partially reported (e.g. stated that the data from two studies which measured a particular outcome were not reported and hence could not be included in the meta-analysis of that outcome). By “synthesis” we mean either a narrative synthesis/summary or meta-analysis of the results. We also recorded whether review authors used any of the following statistical methods to explore whether a meta-analysis was robust to outcome non-reporting bias: the bound for outcome non-reporting bias developed by Williamson et al. [14], the multivariate meta-analysis approach developed by Kirkham et al. [15], or the model-based correction developed by Copas et al. [16]. All data extraction and classification was undertaken by one author (MJP).

### Statistical analyses

The analysis was mostly descriptive, with dichotomous variables (e.g. trial rated at high risk of bias or not) summarised using frequencies and percentages and continuous variables (e.g. number of included trials per review) summarised using medians with interquartile ranges (IQRs). We used the chi-squared test for differences in proportions to explore whether acknowledgements that data were missing from the synthesis of an outcome differed according to reviewer-perceived importance of the outcome.

## Results

### Characteristics of included reviews and trials

We examined 586 reviews including 8434 studies. Reviews included a median of eight studies (IQR 4–16), and addressed a wide range of topics, with 50 Cochrane Review Groups contributing at least one review to the sample. The median number of reviews per Cochrane Review Group was eight (IQR 4–15) (Additional file 1: Table S1).

Of the 8434 included studies, the selective reporting domain was rated as low risk in 4473 (53 %), unclear risk in 2906 (34 %) and high risk in 1055 (13 %). Of the 586 reviews, 239 (41 %) included at least one study rated at high risk of selective reporting bias. In these 239 reviews, a median of 20 % (IQR 10–40 %) of the studies per review were rated at high risk of selective reporting bias.

### Reasons for high risk of selective reporting bias

Across the 1055 studies rated at high risk of selective reporting bias, we identified 89 different reasons provided by review authors to support their judgement. These were classified under nine categories (Table 2; all reasons are listed in Additional file 1: Table S2). The most common reason was concern about outcome non-reporting bias, which was recorded in 819/1055 (78 %) studies. Less common reasons included concern about the documents available for assessment (e.g. “no protocol available”) (59/1055 [6 %]), reporting of only a subset of measurements, analysis methods or subsets of the data (e.g. subscales) that were pre-specified (58/1055 [6 %]), and post hoc reporting of outcomes, measurements, analysis methods or subsets of the data (56/1055 [5 %]). We considered a small proportion of review authors’ reasons to be irrelevant to the selective reporting domain (73/1055 [7 %]); for example, authors stated that not all randomised participants were included in the analysis or that blinding of participants was unclear. The reason for the high-risk judgement was considered unclear for 69/1055 (7 %) studies (e.g. review authors stated that “All outcomes were reported”).

**Table 2:** Frequency of reasons for judgements of high risk of selective reporting bias

Reason	Number (%^a^) of 1055 studies
Concerns about outcome non-reporting bias	819 (78)
Not all of the study’s pre-specified outcomes have been reported	387 (37)
One or more outcomes of interest in the review are partially reported so that they cannot be entered in a meta-analysis	364 (35)
The study report fails to include results for a key outcome that would be expected to have been reported for such a study	188 (18)
Concerns about the documents available for assessment (e.g. no protocol was available or the only available report is a conference abstract)	59 (6)
Concerns about reporting of only a subset of measurements, analysis methods or subsets of the data that were pre-specified (e.g. data were reported for only some of the pre-specified time points)	58 (6)
Concerns about post-hoc reporting of outcomes, measurements, analysis methods or subsets of the data (e.g. one or more reported outcomes were not pre-specified in a protocol or trial registry)	56 (5)
Concerns about how outcome data were analysed (e.g. a continuous/ordinal outcome was dichotomised or adjusted effect estimates were not reported)	28 (3)
Concerns about discrepant reporting (e.g. outcome data differed across multiple reports for a particular study)	9 (1)
Other concerns (e.g. only adverse events occurring in at least 5 % of participants were reported, trialists emphasised statistically significant results even though these were less relevant/secondary outcomes)	31 (3)
Concerns that are not relevant to the selective reporting domain (e.g. not all randomised participants were analysed, baseline data were not reported, blinding of participants was unclear)	73 (7)
Unclear reason (e.g. stated that “All pre-specified outcomes were reported” or no reason stated)	69 (7)

^a^Percentages do not sum to 100 as some trials had more than one reason for a high-risk judgement. Review authors stated one reason in the majority of cases (817/1055, 77 %), two reasons for 209/1055 (20 %) studies and three reasons for 29/1055 (3 %) studies

Review authors did not always describe in the RoB table the specific outcome that was selectively reported. Of the 387 studies rated at high risk due to non-reporting, the non-reported outcome was specified in 326 (84 %). Of the 364 studies rated at high risk due to partial reporting, the partially reported outcome was specified in 222 (61 %). And of the remaining studies rated at high risk due to another reason (*n* = 571), the outcome of concern was specified for only 282 (49 %).

### Acknowledging missing data in the synthesis of an outcome

At least one study was rated at high risk of outcome non-reporting bias in 181/586 (31 %) reviews; we examined a random sample of 100 of these reviews. The 100 reviews addressed various health conditions managed by 33 of the 50 Cochrane Review Groups (Additional file 1: Table S1). A median of 20 % (IQR 10–40 %) of the studies per review were rated at high risk of outcome non-reporting bias (Table 3). In 79 (79 %) reviews, the outcomes that were not/partially reported were specified in the RoB tables; 27 reviews clearly described one outcome that was not/partially reported while 52 described more than one outcome. At least one of the non-/partially reported outcomes was considered a primary review outcome in 52/79 (66 %) reviews. In addition, in 51/79 (65 %) reviews, a meta-analysis was performed on at least one outcome that was not/partially reported in some studies (using data from studies that completely reported the outcome).

**Table 3:** Characteristics of the random sample of reviews with at least one included study rated at high risk of outcome non-reporting bias

Characteristics	Number (%), of *n* = 100
Number of included studies	
Total number of studies included in review, median (IQR)	13 (7–32)
Number of studies per review at high risk of outcome non-reporting bias, Median (IQR)	2 (1–5)
Percentage of studies per review at high risk of outcome non-reporting bias, Median (IQR)	20 (11–39)
High risk outcome(s) stated in the risk of bias table	
One non-/partially reported outcome clearly specified	27 (27)
More than one non-/partially reported outcome clearly specified	52 (52)
No outcome specified (e.g. only stated that “Some outcomes were not reported”)	21 (21)
Reviewer-perceived importance of high-risk outcome(s)	
At least one was a primary review outcome	52 (66)^a^
All were secondary review outcomes	27 (34)^a^
Synthesis of high-risk outcome(s)	
At least one outcome was synthesised in a meta-analysis (based on data from studies that completely reported the outcome)	51 (65)^a^
All outcome(s) were synthesised/summarised narratively	28 (35)^a^
Location in the review where the synthesis of at least one high-risk outcome was reported	
Main text	79 (100)^a^
Abstract	63 (80)^a^
Summary of Findings table	47 (59)^a^

^a^The denominator is 79 because 21 reviews did not specify in the risk of bias table the outcome that was not/partially reported

We were unable to assess whether authors of 21 reviews acknowledged that the synthesis of an outcome was missing data that were not/partially reported, because the non-/partially reported outcome was not specified in the RoB table. In the remaining 79 reviews, few included any statement in either the main text (24/79 [30 %]), abstract (11/63 [17 %]) or Summary of Findings table (9/47 [19 %]) that data were missing from a synthesis (Table 4; see individual comments of Additional file 1: Table S3). However, review authors were more likely to acknowledge that data were missing from a synthesis in the main text if the outcome was a primary review outcome (42 % vs 7 %; *P* = 0.0014). Use of a statistical method to explore whether a meta-analysis was robust to outcome non-reporting bias was not reported in any review.

**Table 4:** Number of reviews which acknowledged that the synthesis of an outcome was missing data that were not/partially reported

Location of statement that data was missing from the synthesis	Type of review outcome			Chi-squared test *P* value^b^
	Any outcome, number (%) of reviews^a^	Primary outcomes, number (%) of reviews	Secondary outcomes, number (%) of reviews	
Main text	24/79 (30)	22/52 (42)	2/27 (7)	0.0014
Abstract	11/63 (17)	9/51 (18)	2/12 (17)	0.9358
Summary of Findings table	9/47 (19)	7/35 (20)	2/12 (17)	0.8001

^a^The denominators reflect the number of reviews for which the assessment was possible. For example, only 63 abstracts were assessed because the outcome that was not/partially reported in some studies was only described in the abstract of 63 reviews

^b^Difference in the proportion of reviews with acknowledgement that data were missing from the synthesis of an outcome when the outcome was considered primary versus when the outcome was considered secondary by the review authors

## Discussion

Of 8434 studies included in 586 Cochrane reviews, 53 % were rated at low risk, 34 % were rated at unclear risk and 13 % were rated at high risk of bias due to selective reporting. We classified the reasons for high-risk judgements into nine categories. The most common reason was concern about outcome non-reporting bias (i.e. non-/partial reporting of at least one outcome). Few studies were rated at high risk because of concerns about bias in selection of the reported result (e.g. reporting of only a subset of measurements, analysis methods or subsets of the data that were pre-specified). Review authors often specified in RoB tables the study outcomes that were not reported (84 % of studies), but less frequently specified the outcomes that were partially reported (61 % of studies), or which were concerning for another reason (49 %). At least one study was rated at high risk of outcome non-reporting bias in 31 % of reviews. In a random sample of these reviews, only 30 % incorporated this information when interpreting results, by acknowledging that the synthesis of an outcome was missing data that were not/partially reported.

A strength of our study is that we examined a large cohort of Cochrane reviews, which comprised all reviews published during a specific period (rather than a non-randomly selected sample). Further, collection of data on judgements (low/unclear/high) and supporting text in RoB tables was automated by Cochrane database managers, which removed the potential for errors due to manual data extraction. There are also some limitations. Only one author classified reasons for the judgements of high risk of selective reporting bias, so there is potential for misclassification. However, category labels for many reasons were re-evaluated on multiple occasions, as modifications to categories were made whenever new examples were encountered; this may have reduced the potential for misclassification. Further, it is possible that some studies we examined were included in more than one of the included reviews. Therefore, our estimates of the number of studies at low/unclear/high risk of bias may have double-counted some studies. However, given that Cochrane strives to produce reviews addressing mutually exclusive questions, we suspect that the number of overlapping studies is low. Finally, we only examined Cochrane reviews so our findings may not generalise to non-Cochrane reviews which use the RoB tool.

The percentage of Cochrane reviews in our sample with at least one study suspected of outcome non-reporting bias (31 %) is lower than that observed in previous research. This bias was suspected in at least one study in 34 % of 283 Cochrane reviews published between 2006 and 2007 [2], but only the primary outcome in each review (rather than all outcomes) was assessed. When all outcomes were assessed in 46 Cochrane cystic fibrosis reviews, 100 % of reviews included at least one study suspected of outcome non-reporting bias [17]. Rather than use the risk of bias assessments by Cochrane reviewers, both investigations used a 9-point classification system to assess studies (ORBIT classification [2, 18]), and involved methodologists in the assessment. It is possible that ours is an underestimate of the true extent of the problem of outcome non-reporting bias, because of variation in how review authors interpret the guidance for the RoB tool, and in how Cochrane Review Groups enforce this guidance. In a 2014 survey of managing and coordinating editors of 42 Cochrane Review Groups, only 57 % expected review authors to search for trial protocols as a step in performing the assessment, and only 23 % considered their review authors to be moderately or largely competent in performing assessments [19]. Therefore, estimates of the frequency of biased studies based on routinely collected risk of bias assessments by Cochrane reviewers should be interpreted with caution [20].

Many of the reasons for high risk of bias judgements were poorly articulated in the RoB tables. For example, statements such as “Some outcomes were partially reported” (encountered in 39 % of studies) make it impossible for readers to know which outcomes to interpret with caution unless they retrieve the primary study report. Further, statements suggesting concern about how outcome data were analysed (e.g. “trialists reported change from baseline values” or “trialists reported unadjusted effect estimates”) are incomplete; it is unclear if review authors were concerned that the decision to report these effect estimates was data-driven or because they find such analytic strategies inappropriate in general. Also, rating a study at high risk of bias because “no protocol was available” means readers are left to guess whether the review authors suspect some outcomes are missing from the published report, or that the reported outcome data have been selected on the basis of the results, or both these reasons, or neither. Review authors often failed to acknowledge that a synthesis of an outcome was missing data that were not/partially reported, and this may have occurred for several reasons. It is possible that review authors believe that completing RoB tables is sufficient, without considering that readers may not refer to these tables [21]. Authors may believe readers are likely to ignore any narrative description of the risk of outcome non-reporting bias and instead just focus on the synthesised effect estimate. Further, the Cochrane Handbook currently does not provide a framework to guide review authors to consider the extent of missing outcome data within a synthesis, and whether its absence is likely to have biased the result (that is, the corresponding risk of bias in the systematic review effect estimate).

Developers of future risk of bias tools could address the problems discussed thus far by adopting the following suggestions. We believe that assessments should be directed at specific results rather than at the study as a whole, to account for that fact that risk of bias may not be the same for each result. Further, we propose that tools designed to assess the risk of bias in effect estimates of individual primary studies should assess bias in selection of the reported result but not outcome non-reporting bias. Outcome non-reporting bias could instead be appraised using a different mechanism, such as a tool to assess the risk that a synthesis (rather than an individual primary study) is affected by reporting biases; this tool could also address the risk of bias due to unpublished studies (“publication bias”) [12]. We are currently involved in projects to develop a reporting bias tool for systematic reviews, and to revise the Cochrane risk of bias tool for randomised trials in line with the suggestions outlined above. We anticipate that these initiatives will help review authors derive more appropriate conclusions about the benefits and harms of interventions.

## Conclusions

Our audit of user practice in Cochrane reviews suggests that the assessment of selective reporting in the current RoB tool does not work well. It is not always clear which outcomes were selectively reported or what the corresponding risk of bias is in the synthesis with missing outcome data. New tools that will make it easier for reviewers to convey this information are being developed.

## Abbreviations

IKMD, Cochrane Informatics and Knowledge Management Department; IQR, interquartile range; ORBIT, Outcome Reporting Bias In Trials; RoB, risk of bias

## Additional file

Additional file 1: Supplementary tables. (DOCX 57 kb)
