# Supplementary material for: Rethinking the assessment of risk of bias due to selective reporting: a cross-sectional study
Source: Syst Rev. 2016 Jul 8;5:108. doi: 10.1186/s13643-016-0289-2 (PMC4938957; doi:10.1186/s13643-016-0289-2)
Supplement: Additional file 1: — Supplementary tables. (DOCX 57 kb) [file 13643_2016_289_MOESM1_ESM.docx]

**Additional file 1: Supplementary Tables**

**Table S1. Characteristics of included Cochrane reviews**

| **Characteristics** | **Total sample** | **Random sample*** |
| --- | --- | --- |
|  | **Number (%),**  **of n = 586** | **Number (%), of n = 100** |
| *Issue number* |  |  |
| 1, 2015 | 57 (10) | 10 (10) |
| 2, 2015 | 82 (14) | 9 (9) |
| 3, 2015 | 68 (12) | 16 (16) |
| 4, 2015 | 97 (17) | 14 (14) |
| 5, 2015 | 60 (10) | 12 (12) |
| 6, 2015 | 84 (14) | 13 (13) |
| 7, 2015 | 65 (11) | 13 (13) |
| 8, 2015 | 73 (13) | 13 (13) |
| *Cochrane Review Group* |  |  |
| Gynaecological, Neuro-oncology and Orphan Cancer | 55 (9) | 4 (4) |
| Pregnancy and Childbirth | 51 (9) | 5 (5) |
| Anaesthesia, Critical and Emergency Care | 29 (5) | 0 |
| Pain, Palliative and Supportive Care | 27 (5) | 6 (6) |
| Heart | 26 (4) | 4 (4) |
| Cystic Fibrosis and Genetic Disorders | 21 (4) | 5 (5) |
| Eyes and Vision | 21 (4) | 4 (4) |
| Wounds | 21 (4) | 2 (2) |
| Skin | 20 (3) | 6 (6) |
| Acute Respiratory Infections | 19 (3) | 2 (2) |
| Gynaecology and Fertility | 19 (3) | 7 (7) |
| Airways | 16 (3) | 4 (4) |
| Musculoskeletal | 15 (3) | 3 (3) |
| Neonatal | 15 (3) | 1 (1) |
| Common Mental Disorders | 14 (2) | 6 (6) |
| Vascular | 14 (2) | 1 (1) |
| Kidney and Transplant | 13 (2) | 6 (6) |
| Epilepsy | 12 (2) | 1 (1) |
| Schizophrenia | 12 (2) | 6 (6) |
| Effective Practice and Organisation of Care | 11 (2) | 0 |
| Upper GI and Pancreatic Diseases | 11 (2) | 0 |
| Bone, Joint and Muscle Trauma | 10 (2) | 1 (1) |
| Developmental, Psychosocial and Learning Problems | 10 (2) | 2 (2) |
| Stroke | 9 (2) | 0 |
| Dementia and Cognitive Improvement | 8 (1) | 4 (4) |
| Drugs and Alcohol | 8 (1) | 2 (2) |
| Neuromuscular | 8 (1) | 2 (2) |
| Inflammatory Bowel Disease (IBD) | 7 (1) | 0 |
| Infectious Diseases | 7 (1) | 2 (2) |
| Oral Health | 7 (1) | 2 (2) |
| Back and Neck | 6 (1) | 1 (1) |
| Breast Cancer | 6 (1) | 0 |
| Injuries | 6 (1) | 0 |
| Childhood Cancer | 5 (1) | 0 |
| Haematological Malignancies | 5 (1) | 1 (1) |
| Lung Cancer | 5 (1) | 1 (1) |
| ENT | 4 (1) | 3 (3) |
| Hypertension | 4 (1) | 2 (2) |
| Incontinence | 4 (1) | 1 (1) |
| Colorectal Cancer | 3 (0.5) | 2 (2) |
| Consumers and Communication | 3 (0.5) | 1 (1) |
| Fertility Regulation | 3 (0.5) | 0 |
| Metabolic and Endocrine Disorders | 3 (0.5) | 0 |
| Tobacco Addiction | 3 (0.5) | 0 |
| Work | 3 (0.5) | 0 |
| Hepato-Biliary | 2 (0.3) | 0 |
| Multiple Sclerosis and Rare Diseases of the CNS | 2 (0.3) | 0 |
| HIV/AIDS | 1 (0.2) | 0 |
| Public Health | 1 (0.2) | 0 |
| Urology | 1 (0.2) | 0 |

*Random sample of 100 Cochrane reviews with at least one trial rated at high risk of outcome non-reporting bias (i.e. non- or partial reporting of an outcome)

**Table S2. Frequency of reasons for judgements of high risk of selective reporting bias**

| Reason | Number (%^a^) of 1055 studies |
| --- | --- |
| Concerns about outcome non-reporting bias | **819 (77.6)** |
| Not all of the study’s pre-specified outcomes have been reported | 387 (36.7) |
| One or more outcomes of interest in the review are reported incompletely so that they cannot be entered in a meta-analysis | 364 (34.5) |
| The study report fails to include results for a key outcome that would be expected to have been reported for such a study | 188 (17.8) |
| Concerns about the documents available for assessment | **59 (5.6)** |
| The only available report is a conference abstract | 35 (3.3) |
| No trial protocol or trial registration record is available | 19 (1.8) |
| No statistical analysis plan is available | 2 (0.2) |
| The certificate of analysis is missing | 1 (0.1) |
| The report is not published in a peer-reviewed journal | 1 (0.1) |
| The trial was retrospectively registered | 1 (0.1) |
| Concerns about reporting of only a subset of measurements, analysis methods or subsets of the data that were pre-specified | **58 (5.5)** |
| Data are reported for a subset of the time points pre-specified | 23 (2.2) |
| Outcome data for both periods of treatment were not available for a crossover trial | 17 (1.6) |
| A subset of the pre-specified subgroup analyses were reported | 16 (1.5) |
| Multiple assessors (e.g. patient and investigator) measured an outcome domain but only data for one assessor was reported | 1 (0.1) |
| A subset of the pre-specified subscales were reported | 1 (0.1) |
| Concerns about post-hoc reporting of outcomes, measurements, analysis methods or subsets of the data | **56 (5.3)** |
| One or more reported outcomes were not pre-specified in a protocol or trial registry | 34 (3.2) |
| Analyses were “not reported according to the protocol” | 10 (1.0) |
| The outcome measure/definition was changed post-hoc | 3 (0.3) |
| The analysis population was not pre-specified | 3 (0.3) |
| The primary outcome was not pre-specified | 3 (0.3) |
| The list of adverse events was not pre-specified | 1 (0.1) |
| Post-hoc subgroup analyses were reported | 1 (0.1) |
| Post-hoc analyses of covariance were performed | 1 (0.1) |
| Concerns about how outcome data were analysed | **28 (2.7)** |
| A continuous/ordinal outcome was dichotomised | 4 (0.4) |
| Only within-group comparisons were reported (e.g. baseline versus endpoint) | 4 (0.4) |
| Only change from baseline values were reported | 3 (0.3) |
| An “inappropriate analysis” was performed | 3 (0.3) |
| Adjusted effect estimates were not reported | 3 (0.3) |
| A composite outcome was reported | 2 (0.2) |
| Analysis methods were unclear | 1 (0.1) |
| The analysis was not consistent with randomisation | 1 (0.1) |
| The appropriateness of statistical methods is unclear | 1 (0.1) |
| Data were not converted (no further explanation provided) | 1 (0.1) |
| Not all outcomes were included in regression models | 1 (0.1) |
| Percent change from baseline data were reported | 1 (0.1) |
| An “unusual” outcome metric was used | 1 (0.1) |
| Different cut-points for a dichotomous outcome were used at different time points | 1 (0.1) |
| Data at different time points were inappropriately combined in the analysis | 1 (0.1) |
| Concerns about discrepant reporting | **9 (0.9)** |
| Outcome data differed across multiple reports for a particular study | 4 (0.4) |
| Outcome data were discrepant within a report (e.g. result stated in abstract differs to that stated in the main text) | 2 (0.2) |
| Outcome data posted at www.clinicaltrials.gov are different to the data presented in a conference abstract | 1 (0.1) |
| Outcome data were reported in the abstract but not the Results section | 1 (0.1) |
| Outcome labels were switched post-hoc (e.g. from “primary” to “secondary”) | 1 (0.1) |
| Other concerns | **31 (2.9)** |
| A threshold was used to report adverse events (e.g. only events occurring in at least 5% of participants were reported) | 10 (1.0) |
| No primary outcome was specified in the trial publication | 6 (0.6) |
| Trialists emphasised statistically significant results even though these were less relevant/secondary | 3 (0.3) |
| Only one outcome was reported | 2 (0.2) |
| A modified measurement instrument was used | 2 (0.2) |
| Outcome data needed to be extrapolated from figures | 1 (0.1) |
| No outcomes were specified in the Methods section | 1 (0.1) |
| No subsets of data were reported | 1 (0.1) |
| It is unclear when the threshold used to define an outcome was set | 1 (0.1) |
| Outcome data were not reported according to length of treatment | 1 (0.1) |
| P-values were reported for a subset of outcomes | 1 (0.1) |
| An intracluster correlation coefficient (ICC) was not reported | 1 (0.1) |
| A dichotomous outcome was defined using modified criteria | 1 (0.1) |
| Concerns that are not relevant to the selective reporting domain | **73 (6.9)** |
| Outcome data were not based on all participants randomised (i.e. intention-to-treat analysis) | 25 (2.4) |
| Some participants were excluded post-randomisation | 7 (0.7) |
| The number of participants randomised to each group is unclear | 6 (0.6) |
| The measurement scale used is not validated | 4 (0.4) |
| It is unclear how the outcome was measured | 2 (0.2) |
| The duration of follow-up was unclear | 2 (0.2) |
| There were imbalances between groups on an outcome at baseline | 2 (0.2) |
| Baseline characteristics were not reported so it is unclear if groups were comparable at baseline | 2 (0.2) |
| Only a “per-protocol analysis” or “completer analysis” was reported | 2 (0.2) |
| There were six primary outcomes but the sample size calculation was based on only one of these | 2 (0.2) |
| It is unclear how data for patients who died was addressed in the analysis | 1 (0.1) |
| The number of participants in each group was different for each outcome | 1 (0.1) |
| Outcome measures were not clearly described | 1 (0.1) |
| It is unclear if participants were blinded | 1 (0.1) |
| Measurement of the outcome was differential between groups | 1 (0.1) |
| The duration of the trial was not specified | 1 (0.1) |
| The trial stopped early | 1 (0.1) |
| The intervention was poorly described | 1 (0.1) |
| Loss to follow-up was not included in the time-to-event analysis | 1 (0.1) |
| Patient recruitment rates were low | 1 (0.1) |
| The measurement scale used was not peer-reviewed | 1 (0.1) |
| Trialists made a unit of analysis error | 1 (0.1) |
| Baseline characteristics were not compared statistically | 1 (0.1) |
| It is unclear whether an assessment of compliance was carried out | 1 (0.1) |
| It is unclear whether concomitant medications were permitted or whether participants were compliant | 1 (0.1) |
| The placebo used may have caused adverse events | 1 (0.1) |
| Data analysis was performed unblinded | 1 (0.1) |
| A surrogate outcome was used | 1 (0.1) |
| Some participants were analysed in a group different from the one to which they were randomised | 1 (0.1) |
| Unclear reasons | **69 (6.5)** |
| Only stated that there were “No pre-specified outcomes” | 38 (3.6) |
| The reason provided suggests there were no problems with selective reporting (e.g. review authors stated that “All outcomes were reported”) | 16 (1.5) |
| An outcome was “poorly defined” | 9 (0.9) |
| No reason provided | 5 (0.5) |
| Post-hoc outcome changes occurred (not clear whether this refers to omission or addition of outcomes, or changes to outcome labels) | 1 (0.1) |

^a^Percentages do not sum to 100 as some trials had more than one reason for a high risk judgement.

**Table S3. Acknowledgements that the synthesis of an outcome was missing data that were not/partially reported, as stated in the main text, abstract and Summary of findings table of the review**

| **ID** | **Quote** |
| --- | --- |
| ***Comments in the main text (Effect of interventions section)*** | |
| CD003262 | “Participant-assessed changes in rosacea severity at end of study: This was a predefined outcome but was not addressed and we therefore judged the domain for selective reporting as at a high risk of bias” |
| CD004380 | “Three of the 21 studies did not provide data in a way that could be included in meta-analysis.” |
| CD007259 | “Gao 2004a and Gao 2004b did not report data suitable for use in between-group comparisons and the authors did not respond to our requests for additional information.” |
| CD007428 | “We presented only outcome data that were complete and consistently reported in the analyses. We received no response from the authors following our request for further information or data from this trial”  “Pain, assessed using a VAS, and analgesic consumption were recorded for the first five days after surgery. Without providing data, Lee 2007 reported that there were no significant differences in the pain scores between the two groups on the first three postoperative days; however, results from day four and five showed lower pain scores in favour of the Knowles pin group (reported P value = 0.05 on day four; P value = 0.04 on day five).” |
| CD008253 | “The included trial pre-stated an outcome measure of subjective nasal symptoms using a visual analogue score. The individual scores from the raw data were not included in the publication. The overall symptom scores for the combined cohort of participants receiving either betamethasone or placebo drops showed improvement, but there was no significant change for the betamethasone or placebo groups nor any change for individual symptoms.” |
| CD008419 | “In Odkvist 2000, no exact data are given, therefore this study cannot be used in any analysis. In this study, the authors reported a significant improvement in vertigo scores in the active treatment group, established with visual analogue scales. There is no explanation available of the range of the scale, nor of the numbers in the figure, and no baseline figures or P values are reported. In the statistical analysis section, it is stated that student t-tests are used, but no exact data are described.” |
| CD008457 | “De Soet 2002 also measured mutans streptococci, but did not fully report numerical estimates of mutans streptococci levels at the end of the study period and intermediate measurements, just “no significant differences between the two treatment groups” (Table 3)”. |
| CD009436 | “Data regarding inflammatory and total lesion counts from Kwon 2012 were incomplete and unusable in synthesis.” |
| CD009446 | “We could not use in meta-analysis one study with 100 randomised participants, which reported that there were no adverse effects (Ramirez 1993), as the effect estimate was inestimable.” |
| CD009524 | “Clinical global impression of change was assessed in Doody 2008, NCT00912288, CONCERT and CONNECTION using the CIBIC-Plus. However, we were only able to extract results from Doody 2008…Thus, we could not draw conclusions about the efficacy of latrepirdine in terms of changes in clinical impression.” |
| CD009574 | “Due to a lack of data the outcome number of patients with moderate to severe pain could not be assessed.” |
| CD009685 | “AEs were reported in an inconsistent manner between trials and not according to the pre-specified manner required in our protocol. Specifically, data were not available for: patients at risk; patients with multiple events; timing of events; outcomes of events. Therefore, we adopted a descriptive method using the data available to describe the AEs in each trial...No denominators were given for each result, preventing the calculation of the RR and CI.” |
| CD009831 | “Mean Brief Psychiatric Rating Scale (BPRS) total score at endpoint (medium term): Because of missing standard deviations for the trial that contributed data to this outcome, no effect size could be calculated. No validated imputation method could be applied to obtain the missing standard deviations.” |
| CD010138 | “No study reported our outcomes-of-interest, even though the studies surely recorded at least some of our outcomes-of-interest (i.e. mortality, serious adverse events).” |
| CD010182 | “Analyses relating to time to healing were mentioned briefly, with trial authors stating that the cumulative to those in the above analysis. This could not be verified from the trial report since no data or P value for the between-group difference were presented.” |
| CD010443 | “While the Asbee 2009 trial did not report data on GDM that could be included in the review’s meta-analysis, the trial manuscript reported that “No statistically significant differences were noted between the groups in... gestational diabetes mellitus” |
| CD010501 | “Average endpoint ESRS tardive dyskinesia score: The pooled summary result from two of the included studies (Lerner 2001; Lerner 2007) showed a beneficial effect of pyridoxal 5 phosphate when compared with placebo (2 RCTs, n = 60, mean difference (MD) -4.07, 95% CI -6.36 to -1.79). Miodownik 2003 reported the endpoint ESRS tardive dyskinesia scores on an unscaled graph, thereby making the data unable to be used.” |
| CD010591 | “Blom 1997/1998 used a daily record chart to measure symptoms of nasal blockage, clear discharge (runny nose), sneezing, coughing, mucus production and eye irritation. Post-treatment numerical data for each treatment group were not presented, however the authors reported that no significant difference was found for the individual symptoms as well as for the mean sum-score before, during or after therapy; Both Blom1997/1998 and Ciabatti 2009 employed a daily record chart. However, given the lack of numeral representation of data in Blom 1997/1998, we could not perform a meta-analysis.” |
| CD010743 | “Three studies, all at high risk of bias, compared fluoride toothpastes containing xylitol with fluoride-only toothpastes over 30 to 36 months (Petersson 1991; Sintes 1995; Sintes 2002). One of the studies, analysing 248 children, compared low-fluoride plus 3% xylitol (daily dosage unclear) with low-fluoride, and normal-level fluoride plus 3% xylitol with normal-level fluoride (Petersson 1991). The authors did not report data in a usable format, but found no difference in the number of DFS between any group. The study did not consider any other outcomes. We were able to pool the data fromthe other two studies in a metaanalysis, which revealed that fluoride toothpaste containing 10% xylitol (daily dosage unclear) resulted in a 13% reduction in caries increment for DFS (PF -0.13, 95% CI -0.18 to -0.08, P value < 0.00001, 4216 children analysed)”. |
| CD010766 | "No results were reported with regard to potential differences between the experimental group and the control group in excretion levels of toxic metals during or after phase two." |
| CD010834 | “Three studies (participants = 1044) measured quality of life using the Asthma Quality of Life Questionnaire (AQLQ) (Flood-Page 2007; Haldar 2009; Pavord 2012).One study noted that there was no significant difference but did not provide any data (Flood-Page 2007).” |
| CD010952 | “The eight remaining trials could not be included in the meta-analysis due to the study design, as these were either not RCTs or not quasi- RCTs (Boersma 1976; Poddubnyy 2012), or because the authors did not provide the number of participants per treatment arm  (Ansell 1978; Carcassi 1990; Lehtinen 1984; Sydnes 1981), or there was no fitting comparison (Wanders 2005) or the outcomes were presented in a way that we could not extract quantitative data (Muller-Fassbender 1985). Of the 31 trials that could be included in the meta-analysis, only 19 studies provided data for any of the efficacy variables.” |
| CD011735 | “We analyzed six studies with 244 participants for reduction of depression scores. SSRIs were statistically and clinically significant superior over placebo (P = 0.005) (relative improvement 30.4%); SMD -0.39, 95% CI -0.65 to -0.14. The effect size was small according to Cohen’s categories. We could not enter one study into the meta-analysis: paroxetine was not statistically superior (P = 0.08) over placebo (Patkar 2007). The quality of evidence was very low” |
| CD011777 | “Two trials reported outcome data for depression (Verkes 1998; Hirsch 1982). In Verkes 1998, however, no numerical data were reported. Instead the trial authors state there was “...no significant treatment effect...” for this outcome (p.545). Also, although mean scores on the HDRS were reported by Hirsch 1982, insufficient information was provided to enable calculation of accompanying SDs via imputation.” |
| ***Comments in the Abstract*** | |
| CD008419 | “The severity of tinnitus and perception of aural fullness were either not measured or inadequate data were provided in the included studies.” |
| CD009436 | “However, although data from 1 of these 2 trials showed benefit of LGLD for reducing inflammatory lesions (MD -7.60, 95% CI -13.52 to -1.68, 43 participants, 1 trial) and total skin lesion counts (MD -8.10, 95% CI -14.89 to -1.31, 43 participants, 1 trial) for people with acne vulgaris, data regarding inflammatory and total lesion counts from the other study were incomplete and unusable in synthesis.” |
| CD009574 | “No trials could be pooled for the outcome ’number of patients with moderate to severe pain’.” |
| CD009685 | “Reporting of adverse events was incomplete, with a suggestion of significant reporting bias...Progression-free survival (PFS) data were not available in the appropriate format for analysis.” |
| CD009900 | “There were insufficient data on adverse events in the other treatment comparisons.” |
| CD010138 | “None of the included studies assessed the clinically important outcomes noted in our protocol” |
| CD010480 | “Adverse effects were adequately reported in only one study” |
| CD010743 | “Four studies reported that there were no adverse effects from any of the interventions. Two studies reported similar rates of adverse effects between study arms. The remaining studies either mentioned adverse effects but did not report any usable data, or did not mention them at all”. |
| CD010834 | “Two studies measured scores fromthe Asthma Quality of Life Questionnaire (AQLQ), which showed a non-significant difference between mepolizumab and placebo (mean difference (MD) 0.21, 95% confidence interval (CI) − 0.01 to 0.44; participants = 682), in the direction favouring mepolizumab. The third study used the St. George’s Respiratory Questionnaire (SGRQ) and found a significant difference between mepolizumab and placebo (MD 6.40, 95% CI 3.15 to 9.65; participants = 576), which indicated a clinically important benefit favouring mepolizumab. A fourth study noted that there was no significant difference but did not provide any data.” |
| CD010861 | “Maternal mortality was rarely reported” |
| CD011345 | “Unfortunately one large study (n = 1453) reporting live birth and pregnancy had not published outcome data by randomised group and therefore could not be analysed.” |
| ***Comments in the Summary of Findings table*** | |
| CD000307 | Explanatory footnote for downgrading evidence: “Reporting bias: strongly suspected - Four studies or fewer reported data for this outcome.” |
| CD003262 | Explanatory footnote for downgrading evidence: “Downgraded one level due to serious imprecision (small sample sizes in the individual studies, pooling not possible due to missing SDs)” |
| CD006506 | Explanatory footnote for downgrading evidence: “For each continuous outcome, there were studies whose results could not be included in this meta-analysis (see Table 2), therefore the evidence provided here does not include all evidence available” |
| CD007803 | Explanatory footnote for downgrading evidence: “Downgraded 1 step for risk of bias: In remission is at risk of selective reporting bias as only five out of 16 studies reported on this important outcome.” |
| CD009574 | Explanatory footnote for downgrading evidence: “No data available” |
| CD010182 | Explanatory footnote for downgrading evidence: “Assessment of time to healing mentioned in RCT report, but estimates not provided” |
| CD010480 | Explanatory footnote for downgrading evidence: “Only one study adequately reported adverse events” |
| CD010861 | Explanatory footnote for downgrading evidence: “Downgraded one level due to risk of bias: several studies did not report important harms; Downgraded one level due to risk of bias: at least 1 study suitable for this comparison was terminated by trial sponsors. This trial had fatigue as a pre-planned outcome. This raises serious concern on the amount of unpublished results which may have been unfavourable to trial sponsors.” |
| CD011565 | Explanatory footnote for downgrading evidence: “Downgraded for risk of bias (-1) primarily due to concerns with selective outcome reporting in a few studies.” |
